# The complex determinants of Alzheimer’s-type dementias

**DOI:** 10.18632/aging.204478

**Published:** 2023-01-04

**Authors:** Bruce M. Cohen, Kai-Christian Sonntag

**Affiliations:** 1 Robertson Steele Professor of Psychiatry, Harvard Medical School, Boston, MA 02115, USA

**Keywords:** Alzheimer’s-type dementias, energy metabolism, NAD, risk factors, intervention targets

Some medical conditions, for example, infectious diseases, have simple causes and respond to single treatments. However, most illnesses, including neuropsychiatric disorders, are complex in nature and require multimodal treatment. Neurodegenerative disorders, in particular, are influenced by multiple biological processes and modified, in turn, by various experiential, environmental, and stochastic factors.

**Figure 1:**
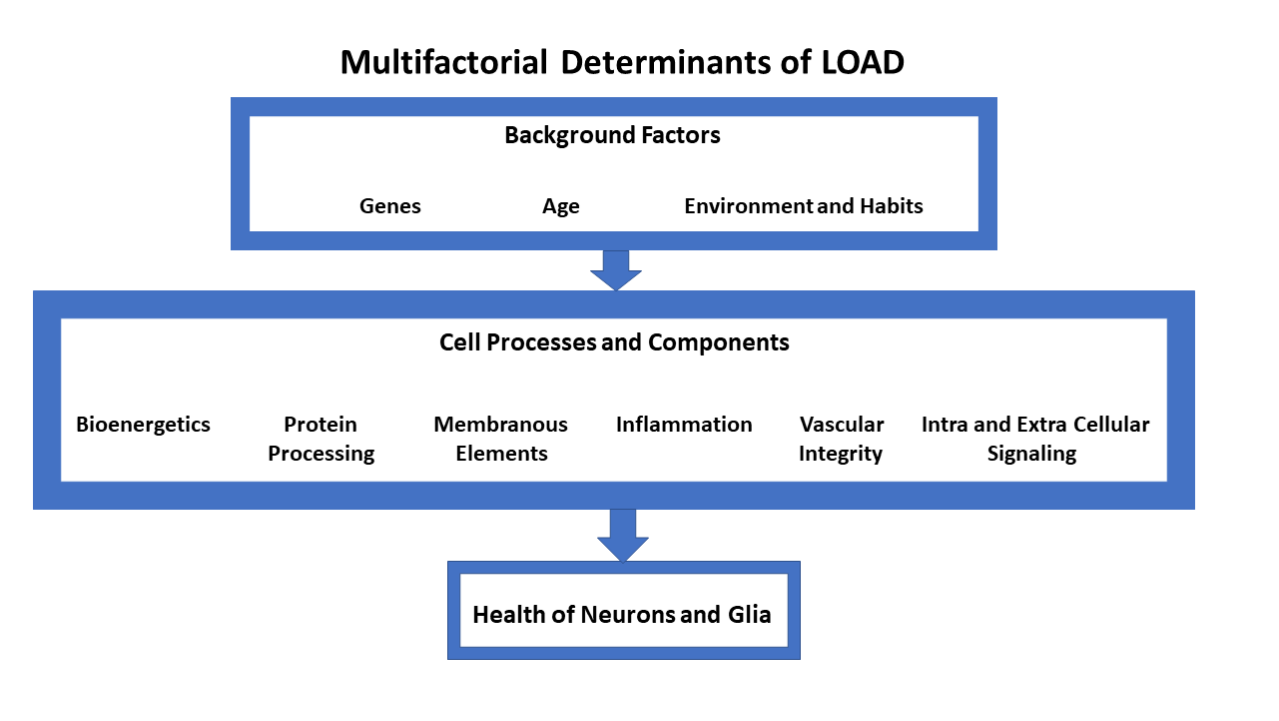
The figure displays the multiple factors that determine the overall risk for late onset Alzheimer’s-type dementias. At each level of the hierarchy shown, the factors interact extensively.

Among neurodegenerative disorders, Alzheimer’s-type dementias are highly variable in etiologies and outcomes. While early-onset Alzheimer’s-type dementias (EOAD) often result from single determinants, late-onset Alzheimer’s-type dementias (LOAD), the most common dementias, are largely the result of multiple interacting factors [1]. Specifically, EOAD-associated single gene variants in APP or Presenilin genes have full penetrance for bearers who reach their 60s. LOAD occurs later than 65, consistent with age being a key risk factor, even if it is not fully determinant. In LOAD, some risk factors are inherent, e.g., the APOE4 gene variant, and others, such as CNS trauma, are experiential. Among the numerous endogenous processes underlying disease expression are alterations in aspects of all of the following: production of proteins, their post-translational modification, and their eventual disposition and digestion; key cellular communication mechanisms, including insulin and neurotransmitter signaling pathways; energy production, through both glycolysis and oxidative phosphorylation; cellular and subcellular membrane amounts, composition, and function; circulatory dynamics and integrity of the blood-brain barrier; inflammatory mechanisms; and the varying activities and interactions of different brain cell types (See Figure 1).

Misprocessed proteins, including beta-amyloids, derived from APP, and hyperphosphorylated tau, which form the pathognomonic plaques and tangles reported by Alois Alzheimer, are always associated with EOAD. And they are usually observed with LOAD. Importantly, however, the source of such misprocessing may not originate with the proteins, themselves, but rather be consequences of abnormalities in other factors. Once formed, these misprocessed proteins are toxic and may lead to more protein misprocessing and cell damage; with that process being hard to restrain. Thus far, efforts targeted at removal of these harmful protein derivatives have not been observed to be safe and effective [2].

By the time abnormal proteins are accumulating, damage to brain circuits and function has already occurred. Intervening earlier, and targeting underlying factors, should be preferable to attempting repairs after plaques and tangles appear.

Among targets for prevention are altered bioenergetic processes observed in LOAD [3]. Bioenergetic abnormalities are associated with most if not all neuropsychiatric disorders, because the brain has a very high energy requirement, 10 to 20-fold that of the body on average. Disruptions in energy metabolism substantially compromise brain health and function. In addition, core bioenergetic pathways, e.g., glycolysis and especially the Krebs cycle, are essential not just for energy production but also for producing substrates for most other cell components, including amino-acids, nucleic acids, membrane constituents, and intracellular and extracellular signaling molecules. Abnormal energy metabolism affects the aging process and all other risk factors and anomalies associated with LOAD, including the development of plaques and tangles.

LOAD-associated bioenergetic alterations are broad-ranging, including anomalies in glucose uptake and metabolism, altered oxidative phosphorylation and Krebs cycle activity, disrupted electron-transport-chain activity, and abnormal NAD (redox) potential. Many of these features are genetically determined and, thus inherent, making them suitable for identification before signs of illness are present [4]. Identifying them in individual patients is practical, and modulating them might delay or prevent LOAD [5].

Among potential targets, NAD is a driving force behind bioenergetic processes. A decrease of total NAD, i.e., the combination of oxidized NAD (NAD+) and reduced NAD (NADH), and changes of the redox-ratio (NAD+/NADH) are striking features in LOAD. An altered redox-ratio suggests imbalances in the production and use of energy. Low total NAD suggests additional impairments in resources for all the enzymatic reactions requiring NAD, which includes most cell processes and pathways, not just bioenergetic pathways.

Decreases in NAD are observed with age as well as in LOAD, and these changes are likely to explain numerous other problems that arise in cell health and function in older people [6]. But unlike the reduction of NAD observed in aging, reduced NAD levels in LOAD are already present during neurodevelopment. Thus, they are likely to be life-long features of metabolism and may be part of a predisposition to an altered aging process underlying LOAD later in life.

NAD is generated from dietary substrates. It has been suggested that dietary supplementation with NAD precursors might be beneficial by boosting NAD levels and, thereby, ameliorating some unwanted aspects of aging [6, 7]. This remains a hypothesis being tested. The supplement most often studied is nicotinamide riboside (NR). We assessed whether supplementation with NR, along with caffeine, which has also been observed to enhance NAD production, would normalize bioenergetic anomalies associated with LOAD [8].

Dermal fibroblasts obtained from individuals with LOAD, and from young and age-matched healthy subjects, were reprogrammed to induced pluripotent stem cells (iPSCs) used to produce neural precursor (NPC)- and astrocyte-like cells in culture [4, 8]. This is the model in which we observed inherent LOAD-associated bioenergetic abnormalities. As expected, treatment with NR and caffeine increased total NAD, but had only modest effects on modulating the redox-ratio. Also, the increases of NAD and their bioenergetic effects were cell-type and cell-origin specific: NAD levels in LOAD NPCs reached levels similar to Control NPCs, but that was not the case for LOAD fibroblasts and astrocytes, and while supplementation initially ameliorated some bioenergetic abnormalities, there were subsequent fluctuations, including some in the opposite direction, with extended treatment. Crucially, the abnormal bioenergetic phenotype associated with LOAD was not normalized.

While NR supplementation may be beneficial in some circumstances, these findings illustrate a key point: LOAD is not a homogeneous or simple entity. It is associated with many individual interacting elements, at the biochemical and cellular levels, and the results of perturbing any one of those elements are often not straightforward or predictable. To stabilize, correct, or prevent complexly determined disorders, it may be necessary to address more than one element.

It is appropriate to propose and test interventions that target one factor associated with LOAD. This is true for continuing efforts to inhibit the production or accumulation of beta-amyloid and phosphorylated tau. However, as studies by our group and others show, many factors contributing to LOAD are inherent and may occur early in the aging process, or even in development, long before plaques and tangles appear. It may be most effective to target the factors underlying the production of abnormal peptides and proteins early in life, well before they build to disruptive levels later in life. And targeting just one underlying factor may not be enough, as no one factor determines the majority of risk for LOAD.

Finally, correcting LOAD-associated changes, such as in energy metabolism, may have broader implications, perhaps improving overall aging as well as preventing the specific deleterious events leading to LOAD.

LOAD appears to be a ‘multi-hit’ disorder, a consequence of the combination of various interacting risk factors, including aging. Some processes will be seen in all or most people with LOAD, and others will be highly individual, requiring a focus on personalized medicine to design and implement therapeutic intervention strategies [5]. Multiprong approaches to LOAD, in those at risk and those with early signs and symptoms, deserve more study. They are likely to be more effective than single target approaches.
